# Differential transcriptional profile of *Corynebacterium pseudotuberculosis* in response to abiotic stresses

**DOI:** 10.1186/1471-2164-15-14

**Published:** 2014-01-09

**Authors:** Anne Cybelle Pinto, Pablo Henrique Caracciolo Gomes de Sá, Rommel T J Ramos, Silvanira Barbosa, Hivana P Melo Barbosa, Adriana Carneiro Ribeiro, Wanderson Marques Silva, Flávia Souza Rocha, Mariana Passos Santana, Thiago Luiz de Paula Castro, Anderson Miyoshi, Maria P C Schneider, Artur Silva, Vasco Azevedo

**Affiliations:** 1 Department of General Biology, Instituto de Ciências Biológicas, Universidade Federal de Minas Gerais, Av. Antônio Carlos, Belo Horizonte 31.270-901, Brazil; 2 Genome and Proteome Network of the State of Pará, Universidade Federal do Pará, R.Augusto Corrêa, Belém 66.075-110, Brazil

**Keywords:** Differential gene expression, Transcripts, RNAseq, SOLID™, Stress, *C. pseudotuberculosis*

## Abstract

**Background:**

The completion of whole-genome sequencing for *Corynebacterium pseudotuberculosis* strain 1002 has contributed to major advances in research aimed at understanding the biology of this microorganism. This bacterium causes significant loss to goat and sheep farmers because it is the causal agent of the infectious disease caseous lymphadenitis, which may lead to outcomes ranging from skin injury to animal death. In the current study, we simulated the conditions experienced by the bacteria during host infection. By sequencing transcripts using the SOLiD^TM^ 3 Plus platform, we identified new targets expected to potentiate the survival and replication of the pathogen in adverse environments. These results may also identify possible candidates useful for the development of vaccines, diagnostic kits or therapies aimed at the reduction of losses in agribusiness.

**Results:**

Under the 3 simulated conditions (acid, osmotic and thermal shock stresses), 474 differentially expressed genes exhibiting at least a 2-fold change in expression levels were identified. Important genes to the infection process were induced, such as those involved in virulence, defence against oxidative stress, adhesion and regulation, and many genes encoded hypothetical proteins, indicating that further investigation of the bacterium is necessary. The data will contribute to a better understanding of the biology of *C. pseudotuberculosis* and to studies investigating strategies to control the disease.

**Conclusions:**

Despite the veterinary importance of *C. pseudotuberculosis*, the bacterium is poorly characterised; therefore, effective treatments for caseous lymphadenitis have been difficult to establish. Through the use of RNAseq, these results provide a better biological understanding of this bacterium, shed light on the most likely survival mechanisms used by this microorganism in adverse environments and identify candidates that may help reduce or even eradicate the problems caused by this disease.

## Background

*Corynebacterium pseudotuberculosis* is a Gram-positive pathogenic bacterium belonging to the class Actinobacteria, which is a member of the *Corynebacterium*, *Mycobacterium*, *Nocardia* and *Rhodococcus* genera (the CMNR group). The CMNR group shares several common characteristics, including (i) the organisation of the cell wall, which is mainly composed of peptidoglycan, arabinogalactan and mycolic acids and (ii) the high G + C content of the genome (47-74%) [1].

The bacterium causes caseous lymphadenitis disease, which affects small ruminants and large animals (such as horses and cattle) worldwide and can infect humans [1]. Therefore, there is an urgent need to control the disease through the development of effective vaccines, therapies and diagnostic kits.

Because *C. pseudotuberculosis* is a facultative intracellular microorganism found preferentially in macrophages in the host, during the infection process, the bacterium is exposed to a number of environmental changes that are far from ideal [2].

After phagocytosis, the phagosome quickly becomes acidic (pH ~ 4.6-5.0) [3] negatively affecting the metabolism and damaging macromolecules in the invading cell. In addition, other intracellular stresses negatively affect the microorganism, including oxidative thermal shock and nitrosative, surface, osmotic and starvation stresses; however, the bacterium manages to escape and persist in the environment [2].

To survive in this environment, the pathogen must mount an immediate and adequate, protective response that is reflected initially by transcriptional changes in specific sets of genes [4]. In this context, sigma factors, which coordinate the expression of these genes under different types of stresses are important [2]; these factors include s*igS* in *E.coli,* which is involved in trehalose synthesis during osmotic stress, with trehalose serving as an important osmoprotectant in this type of stress [4]. In *Mycobacterium tuberculosis*, RT-PCR (real-time polymerase chain reaction) was used to demonstrate the transcriptional profile of 10 sigma factors during the exponential growth phase. The role of these sigma factors was analysed under different conditions of stress, and a number of the factors demonstrated increased expression in response to one kind of stimulus, whereas others responded to more than one stimulus. The resistance to different environmental stresses is associated with the ability of the pathogenic bacteria to survive in the host, and different sigma factors play fundamental roles in the survival of the pathogen. For example, in *M. smegmatis, sigB* is involved in the response to oxidative stress*,* and *sigE* was shown to play a role in the regulation of genes involved in the response to acid stress, thermal shock and sodium dodecyl sulphate (SDS) exposure [5,6].

The functions of sigma factors include involvement in the adaptation to stress, the interaction of the bacterium with the extracellular medium and in a number of cases, with bacterial virulence [7].

For *C. pseudotuberculosis* 1002, there is still no information regarding the role of sigma factors in the regulation of genes involved in bacterial survival throughout the infection process. In addition, few virulence determinants contributing to bacterial survival have been identified. Therefore, investigations related to the control of caseous lymphadenitis have been difficult [1]. To date, the virulence determinants most studied in *C. pseudotuberculosis* infection include the following: the PLD (phospholipase D) protein, an exoprotein that is also considered leukotoxic, contributing to the formation of lesions and the destruction of caprine macrophages during infection [8];the *fagABC* operon and the *fagD* gene, which play a role in the virulence of the bacterium and have been identified as genes involved in iron acquisition [9]; the high concentration of cell wall lipids, which renders the microorganism resistance to digestion by cellular enzymes and allows it to persist as a facultative intracellular parasite [10] and CP40, identified as an immunogenic protein that exhibits proteolytic activity as a serine protease [11].

The availability of the *C. pseudotuberculosis* strain 1002 genome (access number CP001809) has allowed the further investigation and characterisation of the microorganism, which is poorly characterised despite its importance to agribusiness. Therefore, to generate additional information, the transcriptional profile of *C. pseudotuberculosis* was analysed using cDNA sequencing with SOLiD™ 3 plus next-generation technology (Life TechnologiesTM, CA). Using this technology, we investigated the molecular characteristics of the microorganism that allow it to persist in the host and the mechanisms the bacteria use to escape the host immune response. Additionally, in an attempt to contribute to the elimination of caseous lymphadenitis in caprine and ovine populations, we investigated a large number of targets related to *C. pseudotuberculosis* virulence by simulating the specific conditions tolerated by the bacterium when invading the host (i.e., acidic, osmotic and high temperature stresses).

## Results and discussion

The sequencing of cDNA is an attractive technology for the investigation of gene expression in prokaryotic organisms because it provides a high level of coverage and high sensitivity for the detection of transcripts at considerably lower costs compared to traditional methods [12]. Currently, sequencing technology is considered the gold standard for the analysis of gene expression levels [13]. Therefore, because the microorganism can survive environmental changes in the host during infection, we analysed the transcriptional profile of *C. pseudotuberculosis* strain 1002 using RNAseq technology. Acid, osmotic and thermal shock stresses were used to identify genes involved in bacterial tolerance of these unfavourable environments.

**Figure 1:**
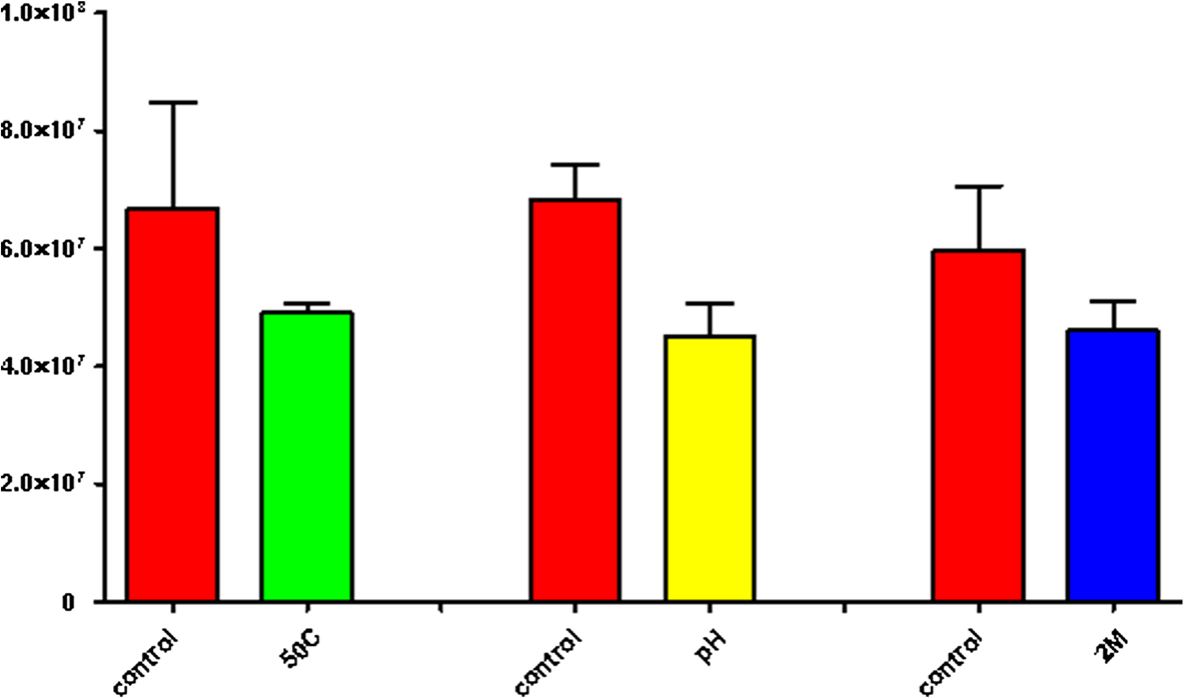
**Number of viable cells under each condition.** The control conditions correspond to approximately 6.7 x 10^7^ cells mL^-1^ of C. *pseudotuberculosis* strain 1002, with the exception of the osmotic stress control, which corresponds to 6.0 x 10^7^ cells mL^-1^. Red, control conditions. Green, thermal shock stress. Yellow, acid stress. Blue, osmotic stress. Figure taken from [16].

Stress-generating agents were applied to the cells at an optical density (OD) of 0.2 (A_600nm_ = 0.2), and the cell viability analysis demonstrated a reduction in replication of approximately 27% under thermal stress, 34% under acid stress and 23% under osmotic stress (Figure 1). A lack of growth or reduced growth is normal during periods of environmental change as the organism attempts to adapt to and physiologically adjust to the new environment [14].

The cDNA samples were sequenced using the SOLiD™ 3 Plus platform, which allowed the analysis of the gene expression profile of the microorganism in the early exponential phase. Using the Bioscope programme, unique readings were mapped on the genome, and gene expression was quantified based on the RPKM (reads per kilobase of coding sequence per million mapped) [15]. Ribosomal transcripts were filtered using Bioscope, and the total number of readings obtained before and after application of the filter is shown in Table 1.

**Table 1:** Number of total readings obtained during sequencing

	**pH**	**2 M**	**50°C**	**Control**
**Gross Data**	17,393,077	18,783,810	21,622,844	25,235,478
**Filtered Ribosomal Transcripts**	9,738,772	9,564,434	9,971,878	9,270,342

Gross data include readings of all transcripts. Filtered ribosomal transcripts refer to the number of readings of transcripts not considered in the analysis. pH- acid stress; 2 M- osmotic stress, 50°C -thermal stress and control -no stress.

Genomic coverage was inferred using the data generated in Bioscope, which represents the level of expression in the RNAseq experiments. The osmotic stress produced the largest number of uniquely mapped transcripts throughout the entire genome, followed by the thermal stress, acid stress and control conditions (Table 2).

**Table 2:** **Number of unique readings mapped in the genome of *C. pseudotuberculosis *1002 and coverage of the transcripts in the genome**

	**2 M**	**50°C**	**pH**	**Control**
**Uniquely mapped readings**	2,016,131	1,633,118	1,764,047	1,650,975
**Genome coverage**	43x	39x	37x	35x

2 M- osmotic stress; 50°C– thermal stress; pH- acid stress and control-no stress.

The automatic annotation of the genome using the Fgenes software (http://www.softberry.com), followed by manual curation of the *C. pseudotuberculosis* strain 1002 genome, identified 2,090 coding regions. From the cDNA sequencing performed using SOLiD^TM^ at the beginning of the exponential phase, 2,055 transcripts active in control were identified (equivalent to 98.32% of the transcribed genome) and 35 genes (1.67%) were considered non-transcripts, exhibiting a RPKM value of 0. Under the different stress conditions, 2,065 (98.80%) transcripts were produced under osmotic stress, 2,063 (98.70%) transcripts under thermal stress and 2,064 (97.76%) under acid stress.

**Figure 2:**
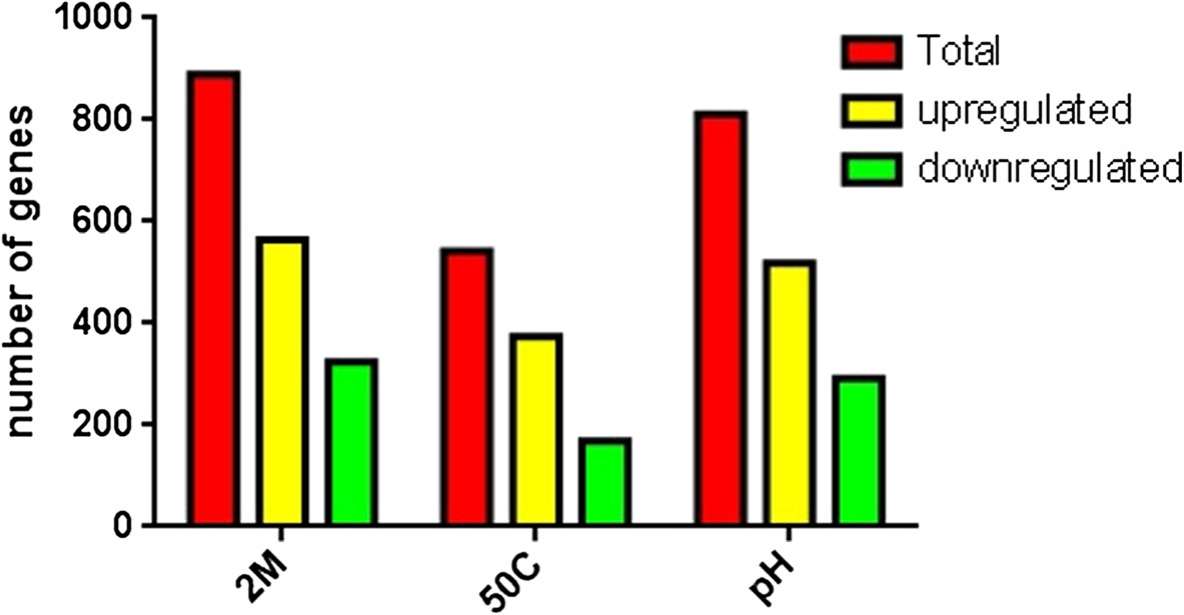
**Number of genes differentially expressed relative to the control.** Red, genes transcribed under each condition (sum of yellow and green). Yellow, genes induced relative to the control. Green, genes repressed relative to the control. 2 M, osmotic stress; 50°C, thermal stress and pH, acid stress.

According to the DEGseq analysis software, of the 2,065 transcripts produced following the osmotic stress, 889 (43.05%) were considered differentially expressed compared to the control (*p-value* <0.001) [16] (Figure 2), 565 of these genes were induced and 324 were repressed. In the thermal stress experiment, 543 (26.32%) transcripts were considered differentially expressed, of which 374 were induced and 169 were repressed. In the acid stress, 811 (39.30%) transcripts were considered differentially expressed, of which 519 were induced and 292 were repressed.

Among the differentially expressed genes, the genes exhibiting a 2-fold change in expression (at least 2x relative to the control) were selected for analysis. The fold-change values were calculated based on the RPKM value between the stress and the control, in which a value greater than one indicated induced gene expression and a value less than one indicated repressed gene expression.

In a previous study reported by our group [17], sequencing of transcripts from *C. pseudotuberculosis* strain Cp31 (biovar equi) was performed using an Ion Torrent platform (Life Technologies). In this study, a comparative analysis between 2 rRNA depletion methodologies was performed, and the transcripts were submitted to *ab initio* assembly. Both transcriptomes were then submitted to gene ontology analysis according to biological processes and molecular functions. Data were obtained only under physiological conditions using brain heart infusion (BHI) media. The authors observed that few transcripts represented genes involved in pathogenicity or the cell adhesion process and concluded that contact with the host may influence the induction of these transcripts.

The present study simulated some of the environmental conditions that the pathogen faces in the host. The cell adhesion process was among those most represented in the conditions described below, and these levels showed greater than a 2-fold change. This result indicated that there were a greater number of transcripts representing genes that participate in the cell adhesion process when compared to the physiological condition. Together, these findings demonstrate that contact with the host most likely influences the transcription of genes essential for bacterial survival.

### Genes induced in the biological processes of the osmotic stress stimulon

**Figure 3:**
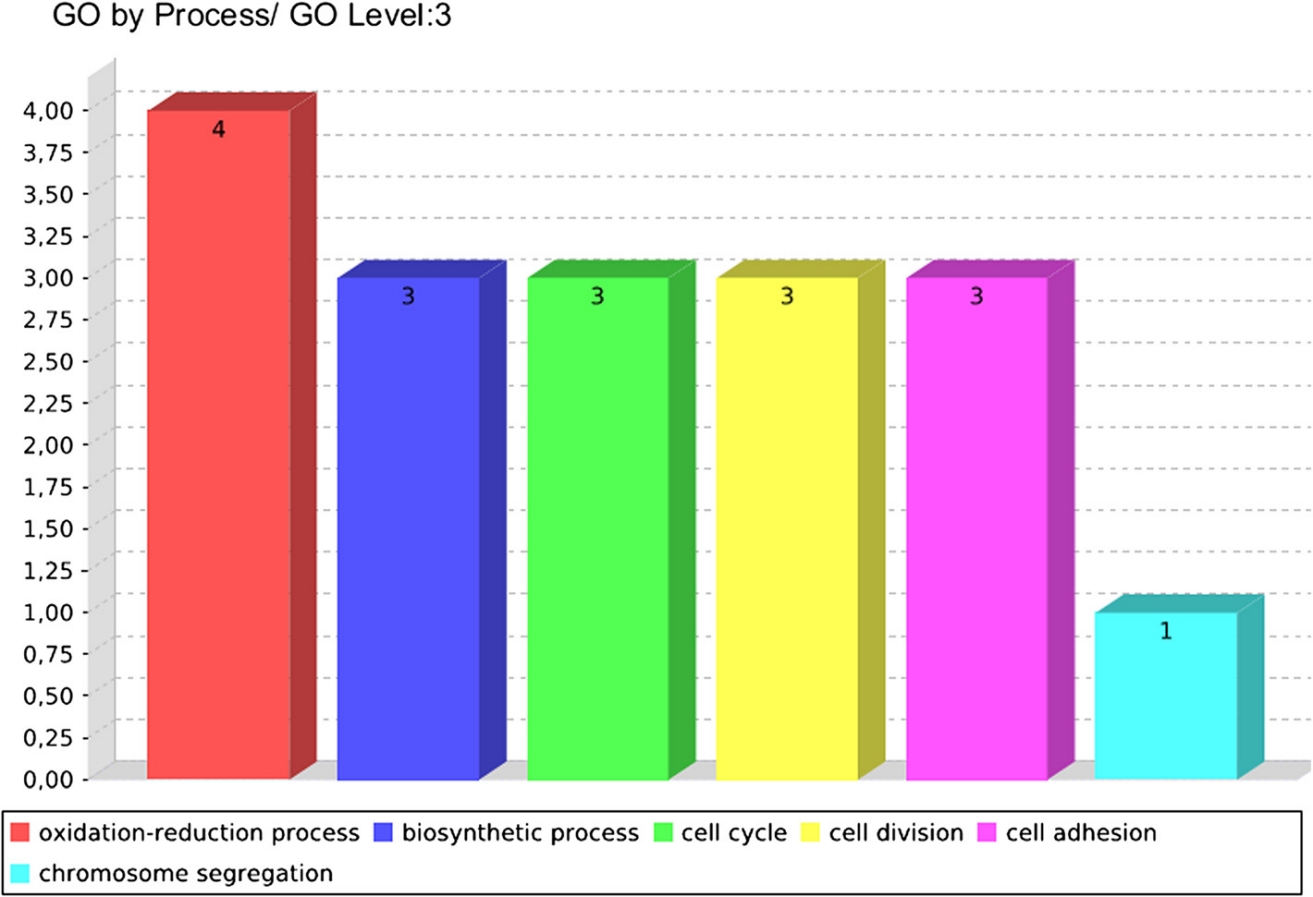
**Biological processes most evident among the genes induced in the osmotic stimulon.** Figure obtained with the CSI program.

The Blast2GO programme was used to identify the biological processes most abundant in the cells under osmotic stress. However, for a more detailed analysis of the processes (Figure 3), the CoreStImulon (CSI) programme [18] was used, which identified the genes present in each of the processes determined in the Blast2GO programme (see Additional file 1: Figure S1).

The majority of the genes induced under osmotic stress were part of the oxidoreduction process, represented by 4 genes (see Additional file 1: Figure S1). Normal aerobic metabolism induces the production of active oxygen molecules, which are increased following exposure to certain environments [19]. The results demonstrated that the growth of the bacterium was reduced but was not interrupted under these conditions; therefore, the bacterium survived in the environment. This observation was confirmed by the biosynthetic process, which was comprised of 3 genes (see Additional file 1: Figure S1), indicating that the bacterium was able to invest energy into the replication process and survived in the unfavourable environment.

The adhesion process, comprising 3 genes, was prominent under osmotic stress. Adhesion is essential for the initiation of the infectious process because the bacterium-host interaction establishes a pathogenic relationship. The Cp1002_0988 gene, encoding a hypothetical protein, is located within 1 of the pathogenicity islands of *C. pseudotuberculosis* 1002, indicating its importance in the development of the disease. Additionally, this gene exhibited a 6.8-fold change in expression levels compared with the control (see Additional file 2: Table S1). The Cp1002_1764 and Cp1002_1765 genes, also identified as encoding hypothetical proteins, exhibited 2.7-fold and 4-fold changes in expression, respectively, compared with the control.

Pathogenic bacteria have developed highly sophisticated signal transduction systems that control the coordinated expression of a number of virulence determinants in response to environmental stresses, and changes in osmolarity contribute to the expression of the genes [20]; therefore, further studies to identify the proteins encoded by these genes and to evaluate the true contribution of these proteins will be necessary.

### Genes induced in the biological processes of the acid stress stimulon

**Figure 4:**
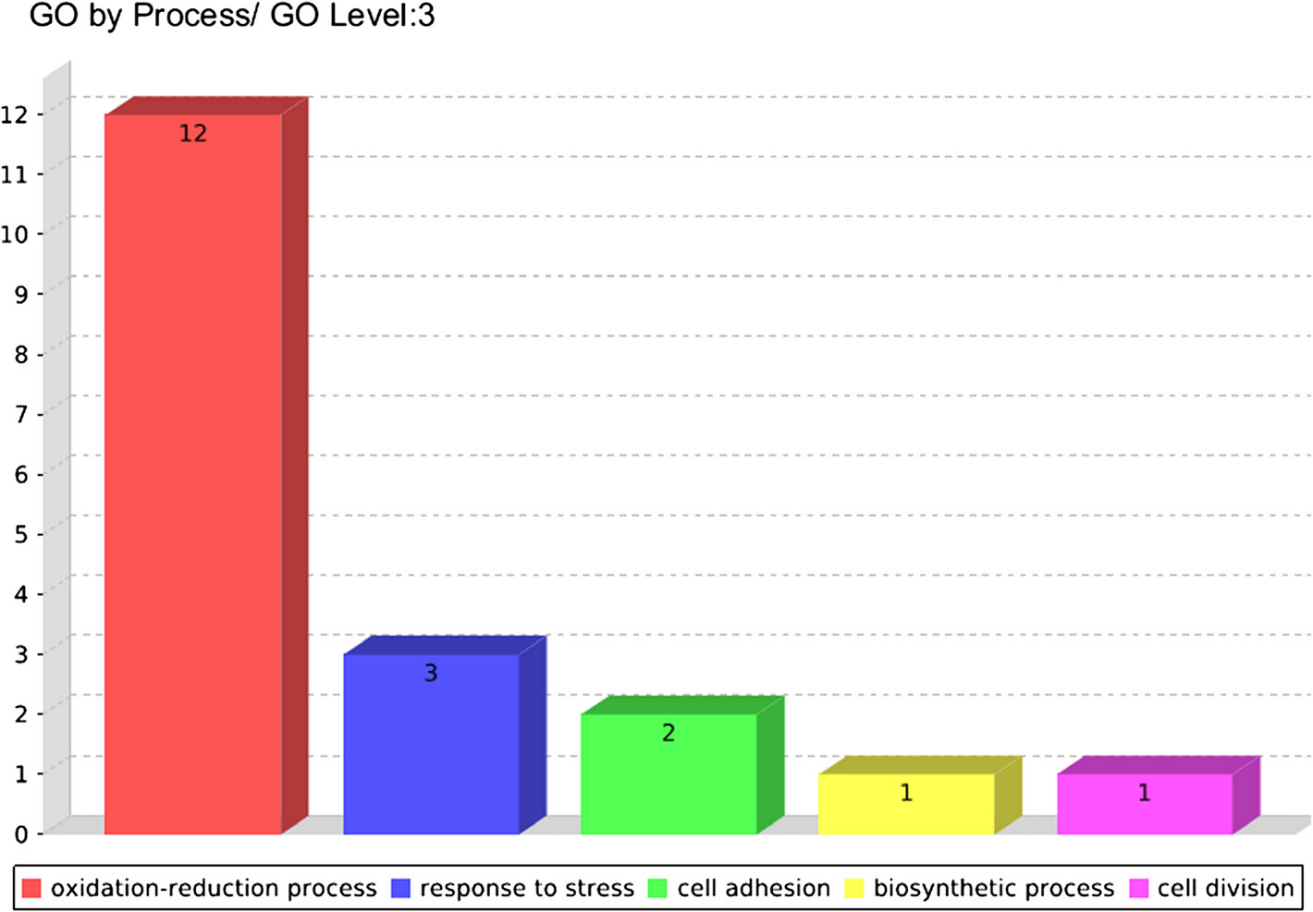
**Biological processes most evident among the genes induced in the acid stimulon.** Figure obtained using the CSI program.

Under acid stress conditions, the induction of genes involved in the processes of cellular adhesion and oxidoreduction in response to stress were of paramount importance because they are associated with virulence (Figure 4) (see Additional file 3: Figure S2). The adhesion processes were composed of hypothetical proteins, 1 of which was characterised as a secreted protein containing an LPxTG domain, which may be an important vaccine candidate. Through analysing the genes that made up the cellular oxidoreduction process, we observed the presence of genes with functions that appeared essential for the persistence of the bacterium in this environment.

The Cp1002_2043 gene exhibited a 7.8-fold change in expression (see Additional file 4: Table S2). This gene, which may encode the Dps protein, was linked to the processes of stress response and cellular oxidoreduction (see Additional file 3: Figure S2). This protein protects the organism against oxidative stress because it stores iron in a bioavailable form, reducing the possibility of the production of reactive oxygen molecules. Under acidic conditions, the production of molecules that produce reactive oxygen species is increased. Reports have demonstrated that in cells in which the pH is decreased, the ratio of HOO^–^ to O_2_^–^ increases [19], increasing the chance of producing hydrogen peroxide (H_2_O_2_). Therefore, the increased number of genes constituting the cellular oxidoreduction process is justified. Another important gene in the oxidoreduction process was Cp1002_0173, which exhibited a 4-fold change in expression and is presumed to encode a catalase that plays a role in reducing the concentration of H_2_O_2_ in the cell.

The mechanisms used by the bacterium to resist the damage caused by reactive oxygen species are essential for survival within the macrophage [21]. Therefore, these proteins might promote the survival of the bacterium in the acid medium starting at the early exponential growth phase.

The Cp1002_1192 *msrB* (methionine sulphoxide peptide reductase) gene, which was 1 of the genes present in the oxidoreduction process, exhibited a high (16-fold) change in expression. Because the MsrA protein may be required to maintain the role of adhesins, the high increase in *msrB* expression suggested that the protein contributes to the survival of the pathogen in the host, the resistance to oxidative stress in vitro and the adhesion capability of eukaryotic cells [22]. The methionine (Met) residues in proteins exposed at the cell surface are thought to be involved in capturing reactive oxygen species, and a complex comprised of MsrA and MsrB reduces the oxidised Met residues, removing the reactive oxygen species [23]. Met is the amino acid most sensitive to reactive oxygen species, and the oxidation of the Met residue in a protein alters the protein structure (or prevents translation), drastically affecting the function [24]. There is evidence that only the MsrB domain is present in *C. pseudotuberculosis* 1002. However, the loss of the *msrA-msrB* domain or *msrB* alone in *Helicobacter pylori* resulted in a reduction in virulence in mouse models, likely because of the oxidation of important proteins [25]. Therefore, the process of cellular oxidoreduction may involve genes that contribute predominantly to the maintenance and persistence of the bacterium in media harmful to the cell.

### Genes induced in the biological processes of the thermal shock stimulon

**Figure 5:**
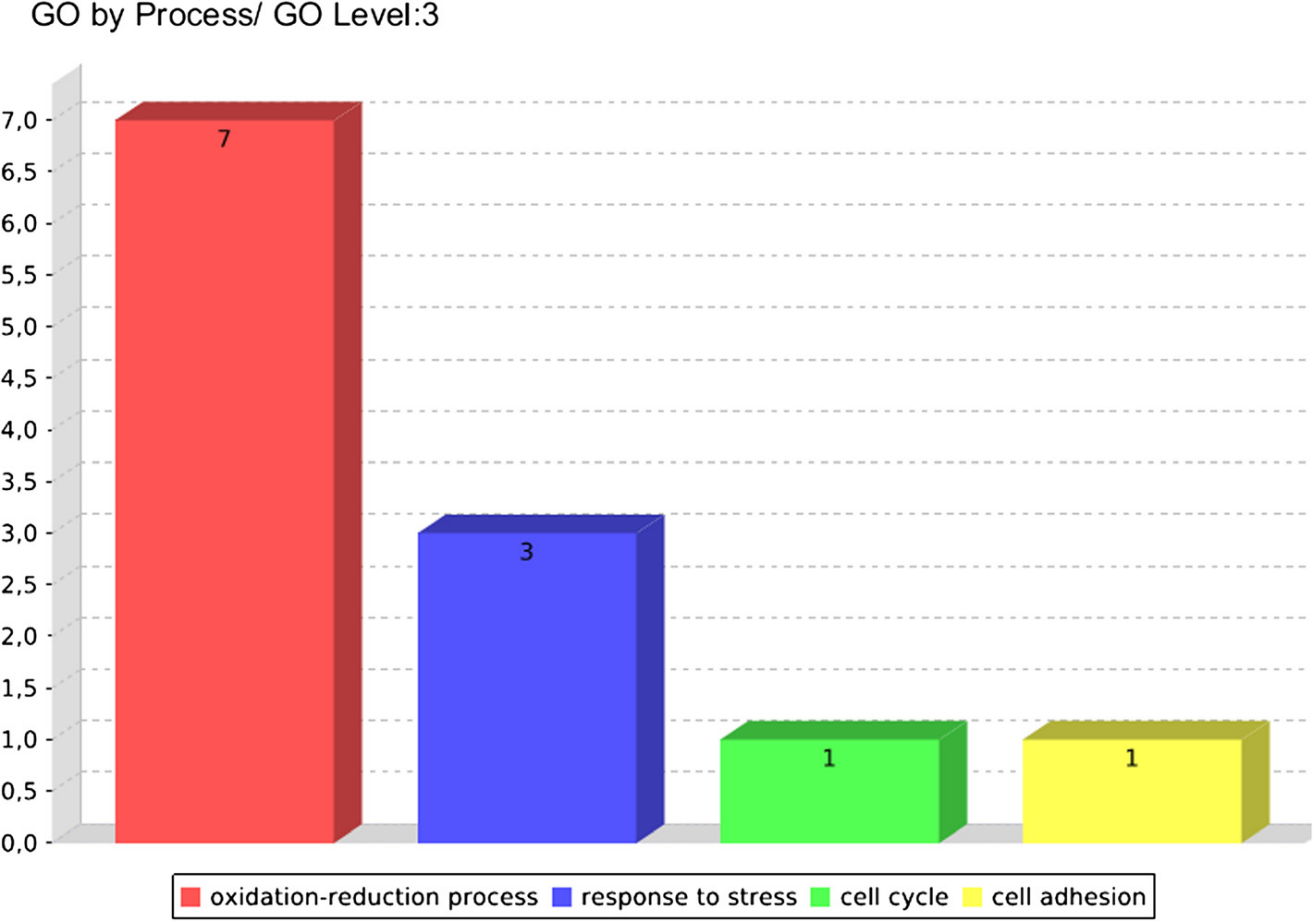
**Biological processes most evident among the genes induced in the thermal stimulon.** Figure obtained using the CSI program.

We analysed the genes involved in the oxidoreduction process under thermal stress. The Cp1002_1785 (*betA*) gene is among the genes that exhibited the highest fold-change in expression values (Figure 5) (see Additional file 5: Figure S3), and the gene may encode choline dehydrogenase. The induced gene exhibited a 5-fold change in expression (See Additional file 6: Table S3) compared to the control. The protein belongs to the oxidoreductase family, which catalyses the oxidation of choline to glycine betaine via the intermediate betaine aldehyde. The protein promotes increased tolerance to hypersalinity and freezing and contributes to the osmotic balance of the cell under stress. The osmoprotectors not only play a role in osmotic balance but also act as effective stabilisers of enzymatic function, providing protection against salinity, high temperatures, freezing, thawing and even dryness [26]. Under conditions of high salinity, the osmoprotectors, together with the transport system, function as virulence factors in certain pathogenic bacteria [20]. Understanding how these elements operate under this condition of stress and their relationship with pathogenesis will be important for future studies.

The adhesion process comprises the Cp1002_1765 gene, which may encode a secreted protein. Cp1002_1765 exhibited a 2-fold change in expression compared with the control, and it is an important candidate for studies related to controlling the disease caseous lymphadenitis.

Among the genes involved in the process of the response to stress, the Cp1002_1895 gene, which may encode a heat shock regulatory protein (HspR), exhibited the largest fold-change in expression (4x relative to the control). This protein acts as a negative regulator of the expression of genes encoding chaperones and proteases in different bacteria under physiological conditions. The heat shock proteins play a key role in cellular metabolism under all growth conditions, monitoring the folding, assembly and translocation of cellular proteins [27]. In *M. tuberculosis*, HspR represses the operon formed by the *dnaK-grpE-dnaJ-hspR* genes through interaction at the HAIR (HspR-associated inverted repeats) region located in the 5′UTR region of the genes. In *C. pseudotuberculosis*, the *hspR* gene is located in the reverse strand, below the *dnaJ, GrpE* and *dnaK* genes, indicative of their regulation by HspR.

It has been suggested that the HspR protein acts as a repressor of other genes linked to virulence/pathogenicity [27]. A study in *M. tuberculosis* demonstrated that the partial disruption of heat-shock regulation influences virulence because the bacterium loses the ability to establish a chronic infection [28]. Mutation of *hspR* in the organism produced increased expression of the DnaK chaperone, which is highly antigenic, resulting in an enhanced immune response in the host. Furthermore, it was demonstrated that DnaK acts as co-repressor for HspR; the activity of HspR is dependent on DnaK. A proposed mechanism for the regulation of *hspR* expression is that under conditions of heat shock, the operon is induced, leading to the increased synthesis of DnaK and HspR. When the concentration of these proteins reaches a critical level, they bind to the promoter of the operon, resulting in repression [29]. To investigate whether this scenario occurred in *C. pseudotuberculosis*, we analysed the gene encoding DnaK and demonstrated that the expression of the gene was induced 2.5-fold relative to the control, which is consistent with the proposed mechanism (see Additional file 6: Table S3).

### Distribution of genes among the simulated conditions

**Figure 6:**
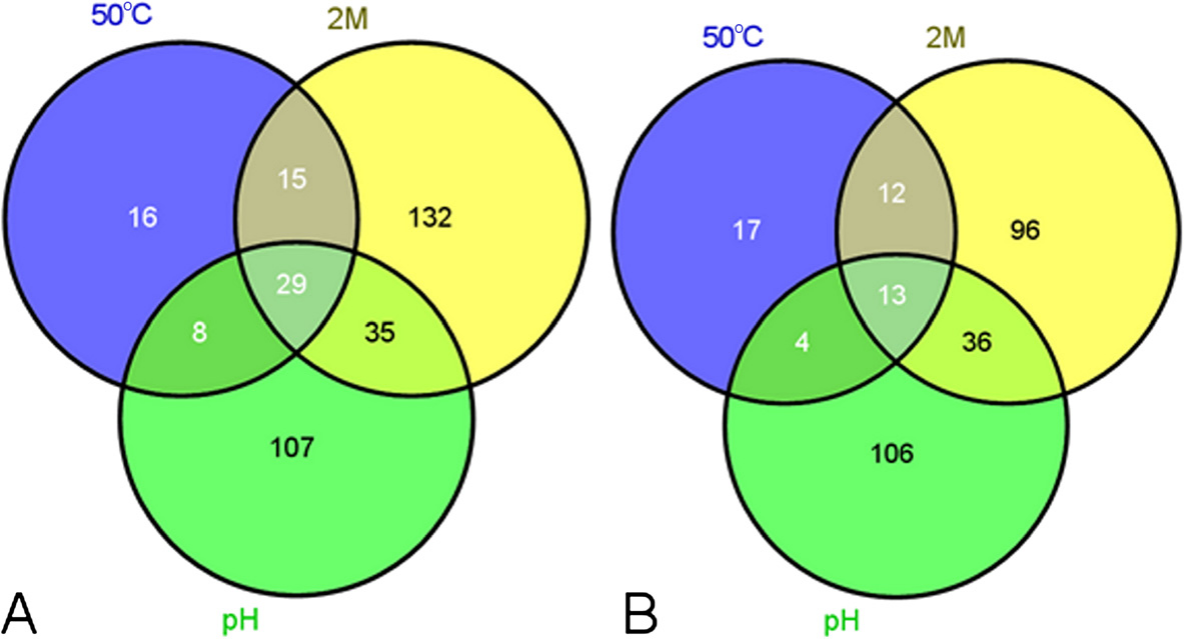
**Venn diagram of the conditions tested.** Distribution of the genes indicating differential expression (**A**-induction and **B**-repression) with at least a 2-fold change relative to the control. 50°C, thermal shock stress; 2 M, osmotic stress and pH, acid stress.

From the Venn diagram (Figure 6) it was possible to observe the distribution of genes per condition, demonstrating 29 genes active in all 3 simulated environments. The analysis of the genes demonstrated that a variation in the fold-change in expression, ranging from 2- to 43-times the control, was observed among the stresses. Of these genes, 48% encoded hypothetical proteins and demonstrated a higher fold-change in expression (see Additional file 7: Table S4) under all conditions. The data demonstrating the high number of unidentified genes reflects the lack of information on *C. pseudotuberculosis*, despite its importance in agribusiness. In the host, the bacterium suffers various stresses simultaneously; therefore, identifying the proteins and their role in the cell is essential for a better understanding of the molecular mechanisms of the bacterium. Presumably, the proteins contribute strongly to the survival and persistence of the bacterium in unfavourable environments; therefore, these proteins are potential candidates for the development of vaccines, diagnostic kits or therapies for caseous lymphadenitis.

Among the shared repressed genes were those encoding proteins related mostly to energy metabolism, sugar transport, amino acids that contribute greatly to the maintenance and replication of the organism in the environment [30], such as *argJ*[31], *nanK*[32], *opp*[33] (see Additional file 8: Table S5) and hypothetical proteins. These data are consistent with the colony-forming unit experiments, which demonstrated a decrease in replication in the stressful environments (Figure 1). A reduction in growth is a survival strategy and occurs in essentially all stressful situations because the organism’s ability to perceive the environment and to modulate the response-controlling mechanisms of resistance, metabolism and other processes [34] which are increased, specific and suitable for the new conditions, is essential.

### Expression of genes encoding sigma factors under simulated conditions

In their natural environment, or in the host during the infection process, the bacterium is exposed to disturbances that require a fast and adaptive response to ensure the survival of the pathogen. Therefore, it is necessary for the bacterium to change the pattern of expression of the genes encoding proteins that directly combat the deleterious nature of the stress [35]. Certain sigma factors play an important and fundamental role in regulating the expression of virulence genes [36]; therefore, it is important to analyse these genes individually.

In the *C. pseudotuberculosis* strain 1002 genome, 8 genes encoding sigma factors were identified, which included the essential sigma factor SigA, non-essential and alternative SigB and 6 alternative factors belonging to the group of extracytoplasmic factors SigC, SigD, SigE, SigH, SigK and SigM, which are dispensable and induced frequently in response to specific conditions, for example, in response to stress.

**Figure 7:**
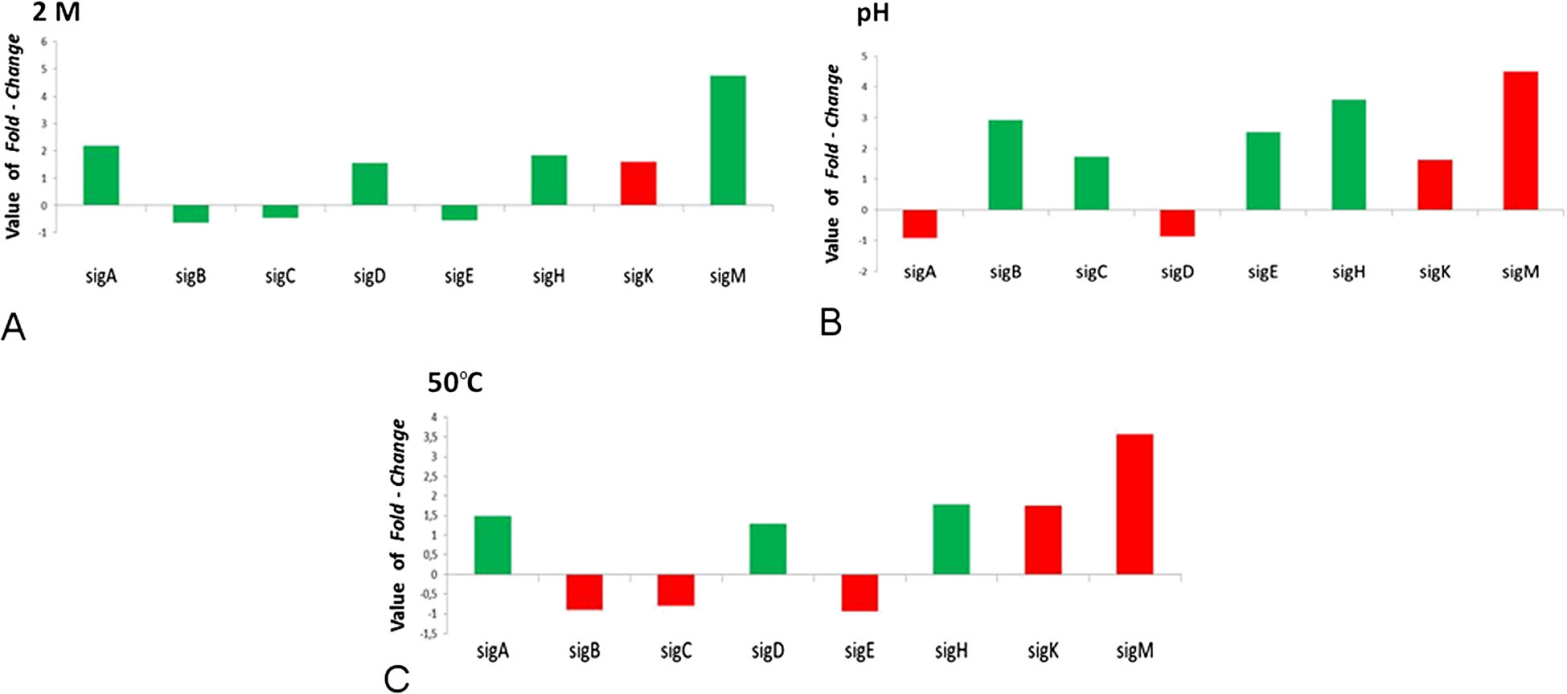
**Expression of genes encoding sigma factors under conditions of simulated stresses.** The fold-change value was based on the ratio between the stress RPKM and the control RPKM. Green columns indicate the differentially expressed genes, independent of the fold-change cutoff established for the analysis, which was at least 2x. Red columns represent the genes not differentially expressed (*p-value* >0.001), established using the DEGseq programme. **a,** Osmotic stress. **b,** Acid stress. **c,** Thermal stress.

Under the osmotic stress conditions, the DEGseq programme demonstrated that only the genes encoding sigma factors A and M were induced (were within the established cutoff of a fold-change of at least 2) (see Additional file 9: Table S6). Under acidic conditions, the genes encoding sigma factors B, E and H were differentially expressed and under thermal stress conditions, the genes encoding sigma factors A, D and H were considered differentially expressed; however, the expression of these genes were below the cutoff for the analysis (Figure 7).

It is possible that *sigA* encodes sigma factor RpoD, alternatively named sigma 70, which promotes the binding of RNA polymerase to specific sites by activating the transcription of most genes essential for exponential growth in *Escherichia coli* (*E. coli*) [37]. In *C. pseudotuberculosis* 1002, the gene contains the 4 domains conserved in the sigma 70 family. Sporadically, σA can act as an alternative sigma factor that is specifically required for the expression of virulence genes. An investigation of the role of *sigA* in *E. coli* in which the *rpoD* gene was induced (from a lack of amino acids and thermal shock), suggested that the protein was involved in the mechanism of recovery from stress [38]. *Streptomyces spp.* contains several homologues of the principal factor that are not essential for replication under normal conditions but appear to play a role under certain growth conditions [39].

Because *sigA* demonstrated changes in expression between the control and the stress conditions in *C. pseudotuberculosis* 1002, it may play a role as an alternative sigma factor, regula [40] ting genes involved in the maintenance of the bacterium.

The gene encoding sigma M was considered differentially expressed only under osmotic stress. This gene may not be expressed preferentially in the early exponential phase. Microarray analysis of *C. glutamicum* in the exponential phase (OD_610nm_ = 0.2 to 0.3) demonstrated that the disruption of *sigM* did not affect the level of transcription of genes induced by thermal shock, which implies that this sigma factor is not involved in the regulation of gene expression in response to this stress [40].

In *M. tuberculosis*, expression of *sigM* occurred in the stationary phase and only under conditions of thermal stress [41]. In *C. glutamicum*, experiments revealed that the deletion of *sigM* caused a reduction in the number of viable cells under conditions of heat shock, cold shock and disulphide (an oxidative stress subtype) stress in the exponential growth phase (OD_600nm_ = 0.7). Furthermore, experiments using real-time PCR demonstrated that the transcription of *sigM* increased significantly after the application of the stresses. These results suggest that sigma factor M is involved in the stress response [42], albeit with a strong indication that the involvement occurs at the late growth phase.

Because there was an increase in the expression of *sigM* under osmotic stress in *C. pseudotuberculosis* 1002, a study of the regulon will be required to identify the genes encoding proteins that assist in the persistence of the bacterium in this hostile environment.

Sigma B is a very common sigma factor in the stress response. The role of the protein in the response to acidity tolerance was established in *Bacillus subtilis*, *Brevibacterium flavum*, *Listeria monocytogenes* and *Staphylococcus aureus*[43-45]. In *B. subtilis*, *SigB* regulates the majority of stress responses, thereby contributing to the transcription of more than100 genes [46]. Another study demonstrated a reformulation of transcription during the infection process and the relevance of sigma B in controlling the expression of virulence genes important for adaptation to the intestinal environment in *L. monocytogenes*[47]. In *L. monocytogenes*, σB contributed to cell survival under different stress conditions [48], and the absence of the factor reduced the ability of the species to invade epithelial cells [49]. It is known that σB contributes to virulence in several Gram-positive pathogens [2], and these observations underscore the need to study the factor separately in *C. pseudotuberculosis* and to identify the regulon that contributes to the survival and escape of the bacterium from the host immune system.

The *sigE* gene, encoding the presumed factor SigE, a member of the σ70 subfamily exhibiting extracytoplasmic function, regulates functions related to perception and response to changes in the periplasm and in the extracytoplasmic environment. In *Haemophilus influenzae,* the expression of *rpoE* increased 102-fold after phagocytosis by macrophages, and the survival of *Haemophilus influenzae* containing a mutated *rpoE* was reduced compared to wild type [50]. According to a study in *Vibrio cholerae*[51], *rpoE* mutants attenuate virulence, and the ability of the bacteria to colonise the mouse intestines is reduced.

According to a study in *C. pseudotuberculosis* 1002 [52], compared with the wild type strain, the mutant 1002 strain is more sensitive to agents that generate nitrosative, acid (pH 5.5) and surface stresses, which indicates the important role SigE plays in the persistence of the bacterium in hostile environments.

The investigation of a mutant *sigE* in *M. tuberculosis* H37Rv [53] demonstrated that the mutant was more sensitive to various environmental stresses, such as thermal shock, SDS and oxidative agents but not to acidity. In a report on *M. Smegmatis*[6], the mutant *sigE* was more sensitive to acidity, hydrogen peroxide, SDS and thermal shock than the wild type. In the *M. tuberculosis* H37Rv study [53], the authors determined that *sigE* is required for the stress response and for the ability to grow and survive inside macrophages. Furthermore, it was suggested that *sigE* influences the level of *sigB* in the cell; however, the expression of *sigB* is not completely influenced by *sigE* because the level of *sigB* was reduced in the mutant strain, whereas the mRNA levels of other sigma factors were not affected.

In *C. pseudotuberculosis* 1002 under acidic conditions, *sigE* was induced and the expression of both *sigE* and *sigB* was significant, whereas under conditions where the mRNA level of *sigE* was not considered significant, the *sigB* expression was also not significant. These sigma factors may influence each other at this exponential phase; however, studies that are more specific will be necessary to verify this hypothesis.

The *sigH* gene, which encodes the extracytoplasmic sigma factor H, was induced (within the established cutoff value) only under the acidic conditions. In *M. tuberculosis,* the *sigH* factor is involved in the response to different stresses and it has been suggested that it regulates the expression of genes involved in the intracellular survival of the microorganism. Additionally, proteins that are part of the *sigH* facto regulon may interact with the host’s immune system, modulating its response [54]. This observation suggests that at the beginning of the *C. pseudotuberculosis* 1002 replication process, the expression of the *sigH* gene and its regulon are required for the bacterium to persist in an acidic environment.

In *C. glutamicum*, a mutation in the *sigH* gene blocked the transcription of *sigM*, and the identification of the *sigH* promoter upstream of *sigM* implied that the factor is under the direct transcriptional control of *sigH*[42]. It was determined that in *C. pseudotuberculosis* 1002, when *sigH* expression increased under conditions of stress, *sigM* expression also increased, although the *sigM* transcripts did not exhibit significant induction during this growth phase. These results suggest that the *sigH* gene responded to an environment similar to that encountered by the bacterium in the host, highlighting the importance of identifying the regulon and the genes responsible for the persistence of the bacterium in the environment.

### Identification of non-coding RNA

The prediction using the RFAM programme identified 5 non-coding RNAs (ncRNAs) in the genome of *C. pseudotuberculosis* strain 1002 (Table 3), including riboswitches (thiamine pyrophosphate [TPP] yybP-ykoY), ncRNA tmRNA (SsrA) and ncRNA mraW.

**Table 3:** **Identification *in silico *of ncRNA in the genome of *Corynebacterium pseudotuberculosis *strain 1002 using the RFAM programme**

**ID**	**Score**	**Strand**	**ncRNA**
RF00023	136.03	+	tmRNA.1
RF00059	57.42	+	TPP.1
RF01747	54.15	-	msiK.1
RF00080	49.16	-	yybP-ykoY.1
RF01746	48.99	-	mraW.1

In most cases, the ncRNAs in bacteria play a role in regulating cellular response during environmental change, which is beneficial to the organism, which needs to adapt quickly and efficiently to these changes and may be impaired by the cell because of the energy cost involved in the expression of a large number of genes. Compared to the synthesis of regulatory proteins, less energy is needed for the synthesis of a small RNA (sRNA) [55]. Among the ncRNAs detected in the genome, riboswitches (elements that control expression in response to various metabolites and can function as sensors or binding sites for a receptor protein that senses a change in the cellular environment) were identified through the analysis of active transcripts. Furthermore, other elements that may be responsible for the synthesis of peptidoglycan, which promotes the expression of genes important under stress conditions [56,57], were identified.

**Figure 8:**
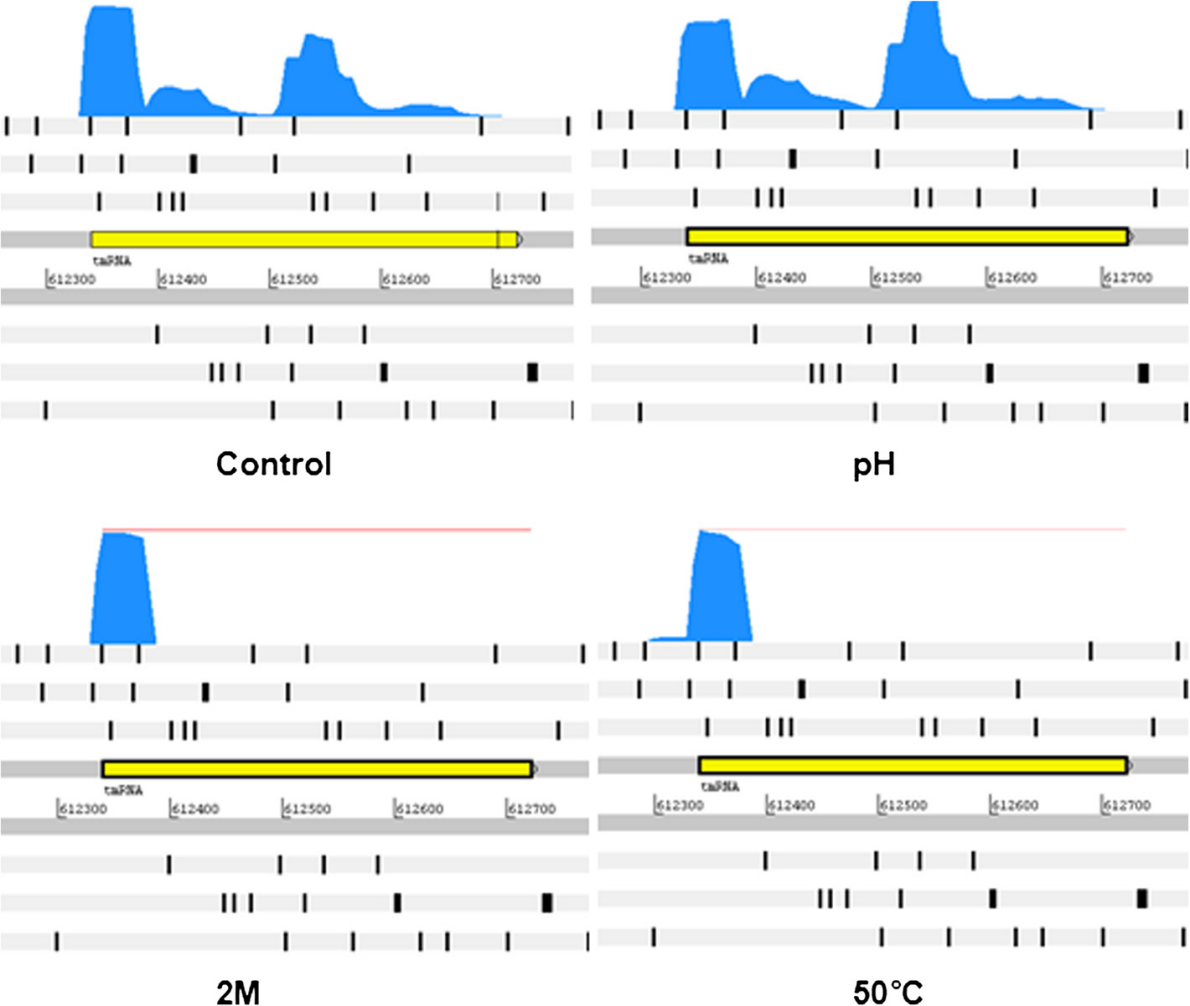
**Coverage of ncRNA transcripts predicted by RFAM.** Control; pH, acid medium; 2 M, osmotic medium and 50°C, thermal shock. Figure obtained in Artemis using the file .BAM, generated in the Bioscope programme.

Coverage of the transcripts was analysed in the predicted element with the highest score. Figure 8 shows the coverage under the control condition and under the stress conditions for the ncRNA tmRNA. This element exhibits dual properties, acting as both messenger RNA and carrier RNA in the rescue of stalled ribosomes [58]. Under acidic and thermal shock conditions, coverage of this ncRNA was slightly superior compared to the control condition. Therefore, it is possible that in the unfavourable environment, because of the reduction in replication, this element is required to attempt the rescue of stalled ribosomes to avoid a loss in protein synthesis, allowing the bacterium to continue to replicate in the environment, albeit at a slower pace. In *Mycoplasma pneumoniae*, *tmRNA* proved essential for growth and in *Salmonella typhimurium*, this element is necessary for survival inside macrophages. It is believed that under conditions of stress, the cells become more sensitive to the activity of *tmRNA*, suggesting the importance of the ability of cells to adapt and survive in different environments [59].

## Conclusion

Next-generation technology allowed the identification of a large number of genes presumed to be required by *C. pseudotuberculosis* 1002 for survival in unfavourable environments, such as acidity, thermal shock and osmotic stress. A number of these genes encode hypothetical proteins, which highlight the need for further investigation of the microorganism. Among the most relevant biological processes identified under all simulated conditions were the processes of adhesion, stress response and oxidoreduction. In these processes, genes involved in the virulence of the organism were affected and should be investigated in more detail to identify the roles they play in the cell, especially inside the host. Furthermore, it is believed that the identification of these genes may contribute to the development of vaccines that are more effective, diagnostic kits and therapies for caseous lymphadenitis.

The expression of sigma factors varied under the different conditions and at the beginning of the exponential phase, these important factors involved in the regulation of genes required for the maintenance of microorganisms under different environmental conditions were observed. Understanding the regulon of the genes would clarify the biology of the organism.

The predicted ncRNAs were identified by the coverage of the transcripts and these factors presumably contribute to the regulation of genes related to the persistence of the bacterium in harmful environments. Further studies will be required to confirm the role of the ncRNAs in escaping the host immune system and contributing to the survival of the bacterium in hostile environments.

The data regarding the expression of induced or repressed genes do not necessarily indicate protein translation; therefore, future experiments will focus on understanding the biology of the transcripts and their products.

## Methods

### Culture conditions: obtaining bacterial cells

*Corynebacterium pseudotuberculosis* strain 1002 was grown in petri dishes containing BHI media (broth composed of (g/L): calf-brain infusion 200.00, beef-heart infusion 250.00, proteose peptone 10.00, dextrose 2.00, sodium chloride 5.00, di-sodium phosphate 2.50 pH 7.4 ± 0.2 at 25°C) at room temperature (RT). One colony was used to prepare the pre-inoculum in 20 mL of BHI media supplemented with 0.05% Tween 80. The culture was grown overnight at 37°C in a shaker at 160 rpm. One millilitre of this pre-inoculum was used to prepare the inoculum in an Erlenmeyer flask containing 100 mL fresh BHI, and this culture was incubated at 37°C at 160 rpm. This preparation was monitored until the beginning of the exponential growth phase (A_600_ = 0.2), which was reached approximately 2.5 hours after the initial inoculation (see Additional file 10: Figure S4).

### Application of stresses

After the culture reached the beginning of the exponential growth phase, the inoculum was divided into 4 50-mL Falcon tubes (1 for each condition), each containing a final volume of 20 mL, and these tubes were then centrifuged for 3 minutes at 8,000 rpm at RT. The pellet was resuspended in fresh BHI specific to each condition. For the acid stress condition, the media was supplemented with hydrochloric acid (which the pH changed to 5). Osmotic stress was achieved with 2 M NaCl, and thermal stress was induced by resuspending the pellet in BHI medium pre-heated to 50°C. In the control condition, bacterial pellets were resuspended in BHI medium at a physiological condition. After the addition of culture media, the tubes were kept in a shaker at 37°C and 160 rpm for 15 minutes, with the exception of the thermal stress sample that was subjected to a temperature of 50°C. An aliquot of each condition was used for decimal dilutions from 10^-1^ to 10^-6^, from which 10^-4^ to 10^-6^ bacteria were seeded in BHI agar, and petri dishes were kept at 37ºC for 48 hours for viability analysis and colony counting (this step was performed in duplicate). The remaining sample was subjected to centrifugation at RT for 3 minutes at 8,000 rpm, and the pellet was resuspended in 2 ml of RNAlater, according to the manufacturer’s instructions.

### RNA extraction

The bacteria suspended in RNAlater® buffer were subjected to total RNA extraction using the ChargeSwitch® total RNA cell kit (Invitrogen, USA) in accordance with the manufacturer’s recommendations, including the following adaptations: after the addition of the lysis buffer (Invitrogen), the material was transferred to 2-mL tubes partially filled with 1-mm diameter glass microbeads (Bertin Technologies). The cells were lysed mechanically using a Prescellys 24 homogeniser, set at 6,500 rpm, for 2 cycles (15 seconds per cycle) with an interval of 30 seconds between the cycles. The samples were centrifuged for 1 minute, and the supernatant transferred to fresh 2-ml tubes and incubated in a dry bath at 60°C for 15 minutes (represents the complete original protocol). DNase was added to eliminate the residual genomic DNA. The elution of the total RNA from the magnetic beads was performed using 100 μL of milli-Q RNase-free water. The amount of total RNA was assessed using a Qubit® 2.0 fluorometer (Invitrogen).

### mRNA enrichment through rRNA depletion

To enrich the mRNA, rRNA from each total RNA sample was removed using the Ribominus™ Transcriptome Isolation kit for yeast and bacteria (Invitrogen, USA), in accordance with the manufacturer’s recommendations. The rRNA-depleted RNA was used for cDNA synthesis using the SOLiD™ Total RNA-Seq kit in accordance with the standard protocol recommended by the manufacturer, and the material was quantified in a Qubit® 2.0 fluorometer (Invitrogen).

### Sequencing in SOLiD™

The depleted RNA was fragmented using RNase III in preparation for amplification of the cDNA library, which was produced by reverse transcription from adapters attached to the ends of the RNA molecules, in accordance with the SOLiD™ Total RNA-Seq kit protocol (Life Technologies™, CA). Next, 6% denaturing polyacrylamide gel electrophoresis was performed and fragments of appropriate sizes (150 to 250 bases) were cut from the gel for cDNA amplification using PCR. Following recommended protocols, the cDNA was purified and the sizes were confirmed using 2% agarose electrophoresis. The PCR amplification in emulsion was performed using primers complementary to the adapters, in accordance with the Applied Biosystems SOLiD™ 3 Plus System Templated Bead Preparation Guide. After amplification, the microspheres were deposited onto slides for sequencing in accordance with the manufacturer’s recommendations. The SOLiD™ 3 Plus system was used to sequence the 50-nucleotide RNA reads.

### Analysis *in silico*

After obtaining the reading files using the SOLiD™ technique, the data were loaded into Bioscope version 1.2.1-5 programme (Life Technologies™, CA), and the gene expression data were obtained and quantified as reads per kilobase of coding sequence per million reads (RPKM) [15]. The DEGseq programme [16] was used to identify differentially expressed genes. The programme uses the output file from Bioscope and the RPKM values as the input file. For differentially expressed genes, a cutoff value of *p* <0.001 was defined. The data from each stimulon were analysed using Blast2GO (http://www.blast2go.com), and the results were exported to CoreStImulon programme [18] to search more rapidly for the genes comprising each biological process defined by Blast2GO.

To predict the non-coding RNA, a similarity search was performed in the RFAM database [60] using the script rfam_scan-1.0.3.pl (http://rfam.sanger.ac.uk/), and sRNAs smaller than 70 bp were discarded to minimise the number of false positives.

Transcriptomic coverage for all sRNAs annotated by RFAM was confirmed by manual curation in the program Artemis using the file BAM.

## Abbreviations

RNA-Seq: high-throughput sequencing of cDNA libraries; PLD: Phospholipase D; RPKM: Reads per kilobase of coding sequence per million mapped; CSI: CoreStImulon; H2O2: Hydrogen peroxide; msrB: peptide methionine sulphoxide reductase; Met: Methionine; cDNA: complementary DNA synthesised from RNA; rRNA: ribosomal RNA; tRNA: transfer RNA; Sig and σ: Sigma factor; spp: species; ncRNA: non-coding RNA; sRNA: small RNA; BHI: Brain heart infusion broth.

## Competing interests

The authors declare there are no competing interests.

## Authors’ contributions

ACP, WMS and FSR performed the bacterial growth and stress application experiments. VA, AS, MPCS and AM offered support for the sequencing of the transcripts, preparation of the reagents and the analysis tools. VA coordinated and directed the research. ACP, SB and HPMB performed the sequencing experiments in SOLiD^TM^. ACP analysed the data. PHCGS, RTJR and ACR offered support in bioinformatics. ACP, MPS and TLPC wrote the manuscript. All authors read and approved the final manuscript.

## Supplementary Material

Additional file 1: Figure S1 **Report of the biological process for the osmotic medium.** The file contains the genes induced in the biological processes in the osmotic medium stimulon, which exhibited fold-change values equal to or greater than 2x relative to the control. Click here for file

Additional file 2: Table S1 **Values of RPKM and fold-change of genes differentially expressed in the osmotic medium**. The table contains the differentially expressed genes in the osmotic medium and their respective RPKM and fold-change values. The column marked TRUE indicates that the genes were considered differentially expressed (*p-value* <0.001), and FALSE indicates that the genes were not considered differentially expressed (*p-value* >0.001). Click here for file

Additional file 3: Figure S2 **Report on the biological process for the acid medium.** The file contains genes induced from the biological processes in the acid medium stimulon, which exhibited fold-change values equal to or greater than 2x relative to the control. Click here for file

Additional file 4: Table S2 **Values of RPKM and fold-change of genes differentially expressed in acid medium**. The table contains the differentially expressed genes in the acid medium and their respective RPKM and fold-change values. The column marked TRUE indicates the genes were considered differentially expressed (*p-value* <0.001), and FALSE indicates that the genes were not considered differentially expressed (*p-value* >0.001). Click here for file

Additional file 5: Figure S3 **Report on the biological process under thermal shock.** The file contains genes induced in the biological processes in the thermal shock stimulon, which exhibited fold-change values equal to or greater than 2x relative to the control. Click here for file

Additional file 6: Table S3 **Values of RPKM and fold-change of genes differentially expressed under thermal shock**. The table contains the differentially expressed genes under thermal shock and their respective RPKM and fold-change values. The column marked TRUE indicates that the genes were considered differentially expressed (*p-value* <0.001), and FALSE indicates that the genes were not considered differentially expressed (*p-value* > 0.001). Click here for file

Additional file 7: Table S4 **Values of RPKM and fold-change of genes considered induced under the 3 conditions.** The table contains the differentially expressed genes induced under the 3 conditions simultaneously and their respective fold-change values and product names. The table contains information for each stress separated by colour. Click here for file

Additional file 8: Table S5 **Values of RPKM and fold-change of genes considered repressed under the 3 conditions.** The table contains the differentially expressed genes repressed under the 3 conditions simultaneously and their respective fold-change values and product names. The table contains information for each stress separated by colour. Click here for file

Additional file 9: Table S6 **Values of RPKM and fold-change of genes encoding sigma factors.** The table contains the RPKM and fold-change values of the genes encoding sigma factors and the genes differentially or not differentially expressed are indicated. The asterisk indicates genes that were not considered differentially expressed. Click here for file

Additional file 10: Figure S4 **Growth Curve**. Plot showing the growth curve of *C. pseudotuberculosis* strain 1002 under the control condition and measured at an optical density of 600 nm. Triangles indicate the density values at each hour. The arrow shows the time-point when the stresses were induced. (OD_600nm_ = 0.2) – indicates the beginning of the exponential phase. Click here for file
