# Supplementary material for: Differential transcriptional profile of Corynebacterium pseudotuberculosis in response to abiotic stresses
Source: BMC Genomics. 2014 Jan 9;15:14. doi: 10.1186/1471-2164-15-14 (PMC3890534; doi:10.1186/1471-2164-15-14)
Supplement: Additional file 1: Figure S1 — Report of the biological process for the osmotic medium. The file contains the genes induced in the biological processes in the osmotic medium stimulon, which exhibited fold-change values equal to or greater than 2x relative to the control. [file 1471-2164-15-14-S1.pdf]

GO by Process/ GO Level:3

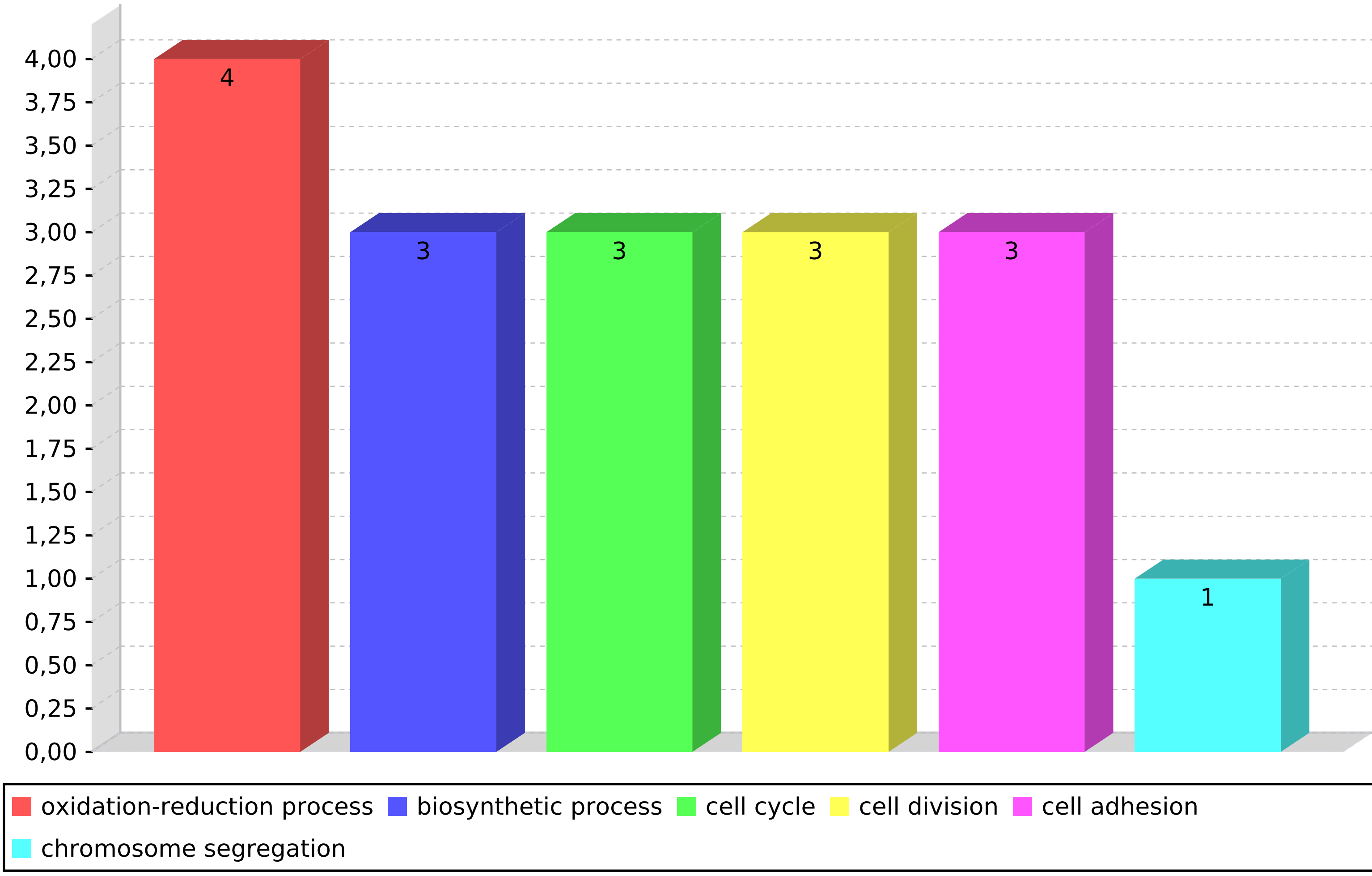

biosynthetic process

CDS	Annotation
Cp1002_0301	glycosyl transferase group 1
Cp1002_0900	isochorismate synthase
Cp1002_1415	mannosyltransferase

cell adhesion

CDS	Annotation
Cp1002_0988	I domain-containing protein
Cp1002_1764	secreted protein
Cp1002_1765	secreted protein

cell cycle

CDS	Annotation
Cp1002_0715	septum formation initiator protein
Cp1002_0965	site-specific tyrosine recombinase
Cp1002_1518	undecaprenyl pyrophosphate synthase

cell division

CDS	Annotation
Cp1002_1397	penicillin-binding protein
Cp1002_0965	site-specific tyrosine recombinase
Cp1002_1518	undecaprenyl pyrophosphate synthase

chromosome segregation

CDS	Annotation
Cp1002_0965	site-specific tyrosine recombinase

oxidation-reduction process

CDS	Annotation
Cp1002_1062	dehydrogenase oxidoreductase
Cp1002_1674	ferritin
Cp1002_0264	pyrroline-5-carboxylate reductase
Cp1002_1930	udp-glucose 6-dehydrogenase
