# Supplementary material for: Differential transcriptional profile of Corynebacterium pseudotuberculosis in response to abiotic stresses
Source: BMC Genomics. 2014 Jan 9;15:14. doi: 10.1186/1471-2164-15-14 (PMC3890534; doi:10.1186/1471-2164-15-14)
Supplement: Additional file 3: Figure S2 — Report on the biological process for the acid medium. The file contains genes induced from the biological processes in the acid medium stimulon, which exhibited fold-change values equal to or greater than 2x relative to the control. [file 1471-2164-15-14-S3.pdf]

GO by Process/ GO Level:3

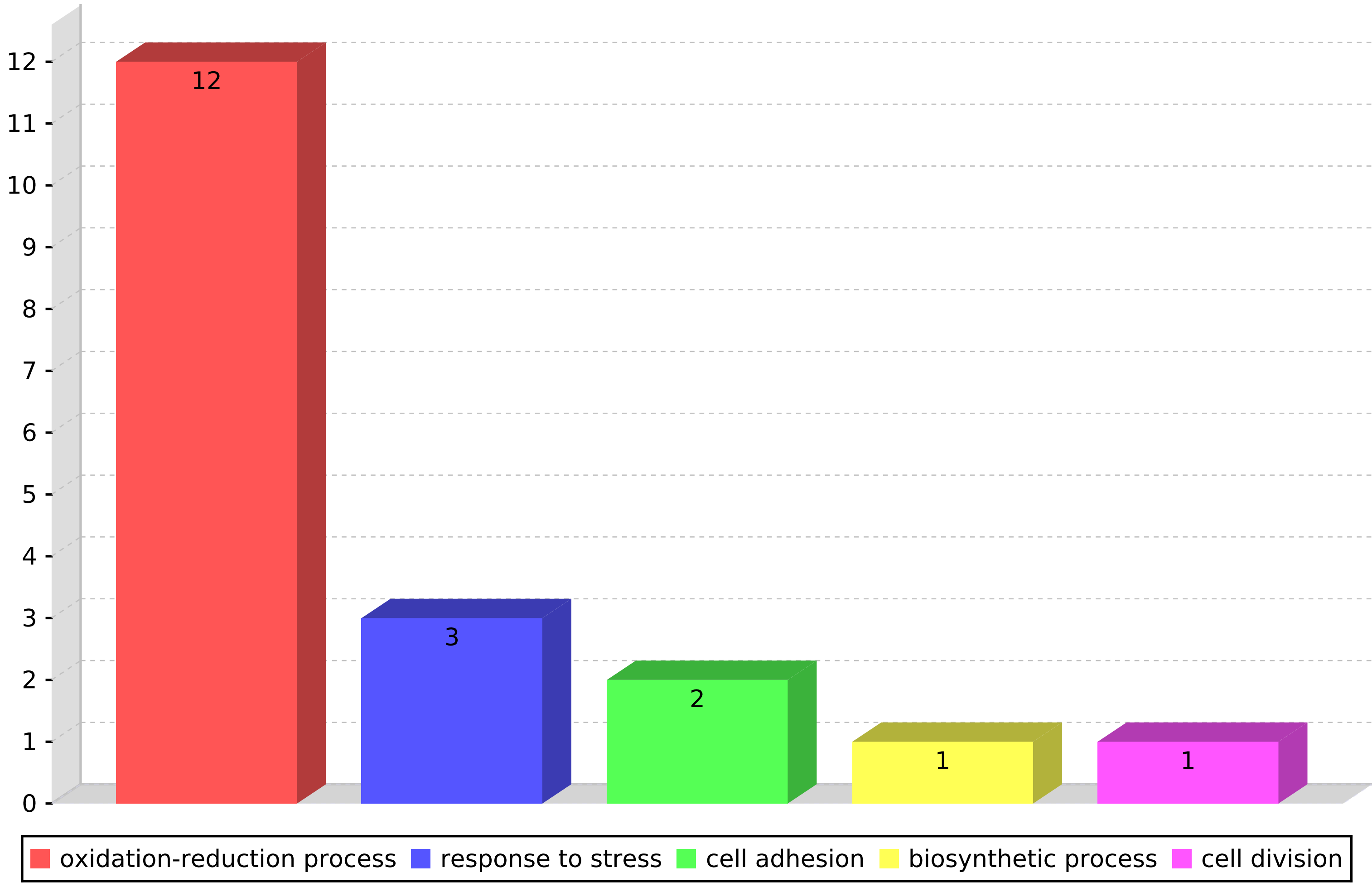

biosynthetic process

CDS	Annotation
Cp1002_0301	glycosyl transferase group 1

cell adhesion

CDS	Annotation
Cp1002_0023	l domain-containing protein
Cp1002_1964	l domain-containing protein

cell division

CDS	Annotation
Cp1002_1400	cell division protein

oxidation-reduction process

CDS	Annotation
Cp1002_1338	alanine dehydrogenase
Cp1002_0084	alpha-ketoglutarate-dependent dioxygenase
Cp1002_0173	catalase
Cp1002_2043	dna protection during starvation protein
Cp1002_1339	glutamate dehydrogenase
Cp1002_1469	inosine 5-monophosphate dehydrogenase
Cp1002_1898	molecular chaperone
Cp1002_1192	peptide methionine sulfoxide reductase
Cp1002_0280	protoporphyrinogen oxidase
Cp1002_1748	pyruvate dehydrogenase
Cp1002_0250	pyruvate formate-lyase activating enzyme
Cp1002_1638	zinc-binding dehydrogenase family protein

response to stress

CDS	Annotation
Cp1002_2043	dna protection during starvation protein
Cp1002_1897	molecular chaperone
Cp1002_1898	molecular chaperone
