# Supplementary material for: Differential transcriptional profile of Corynebacterium pseudotuberculosis in response to abiotic stresses
Source: BMC Genomics. 2014 Jan 9;15:14. doi: 10.1186/1471-2164-15-14 (PMC3890534; doi:10.1186/1471-2164-15-14)
Supplement: Additional file 5: Figure S3 — Report on the biological process under thermal shock. The file contains genes induced in the biological processes in the thermal shock stimulon, which exhibited fold-change values equal to or greater than 2x relative to the control. [file 1471-2164-15-14-S5.pdf]

GO by Process/ GO Level:3

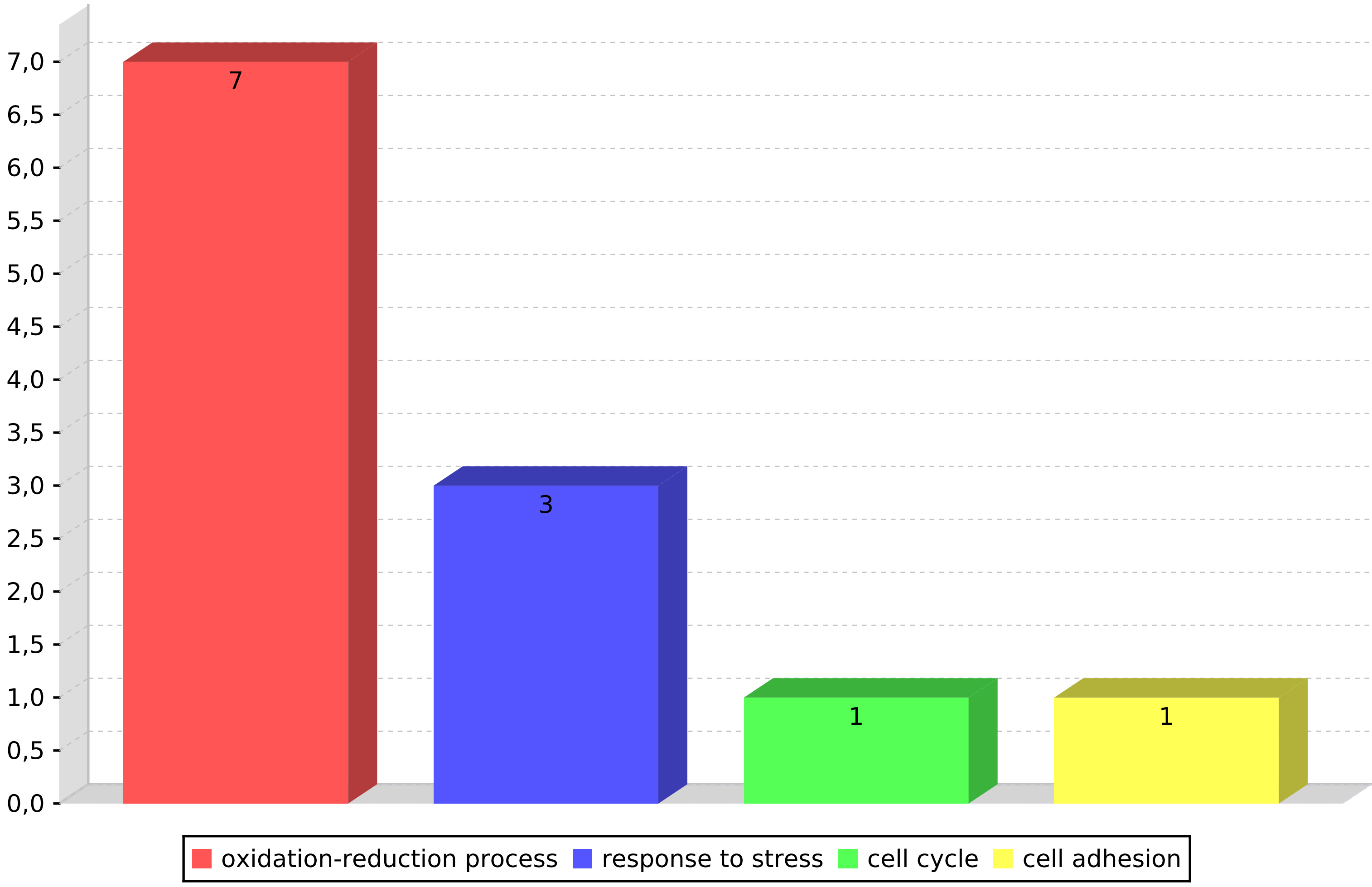

cell adhesion

CDS	Annotation
Cp1002_1765	secreted protein

cell cycle

CDS	Annotation
Cp1002_0715	septum formation initiator protein

oxidation-reduction process

CDS	Annotation
Cp1002_1218	alkyl hydroperoxide reductase
Cp1002_0622	alpha-ketoglutarate-dependent taurine dioxygenase
Cp1002_1783	betaine aldehyde dehydrogenase
Cp1002_1785	choline dehydrogenase
Cp1002_1674	ferritin
Cp1002_1339	glutamate dehydrogenase
Cp1002_1898	molecular chaperone

response to stress

CDS	Annotation
Cp1002_1895	heat shock protein
Cp1002_1897	molecular chaperone
Cp1002_1898	molecular chaperone
