# Supplementary figures and images for: Differential transcriptional profile of Corynebacterium pseudotuberculosis in response to abiotic stresses
Source: BMC Genomics. 2014 Jan 9;15:14. doi: 10.1186/1471-2164-15-14 (PMC3890534; doi:10.1186/1471-2164-15-14)

# Growth Curve

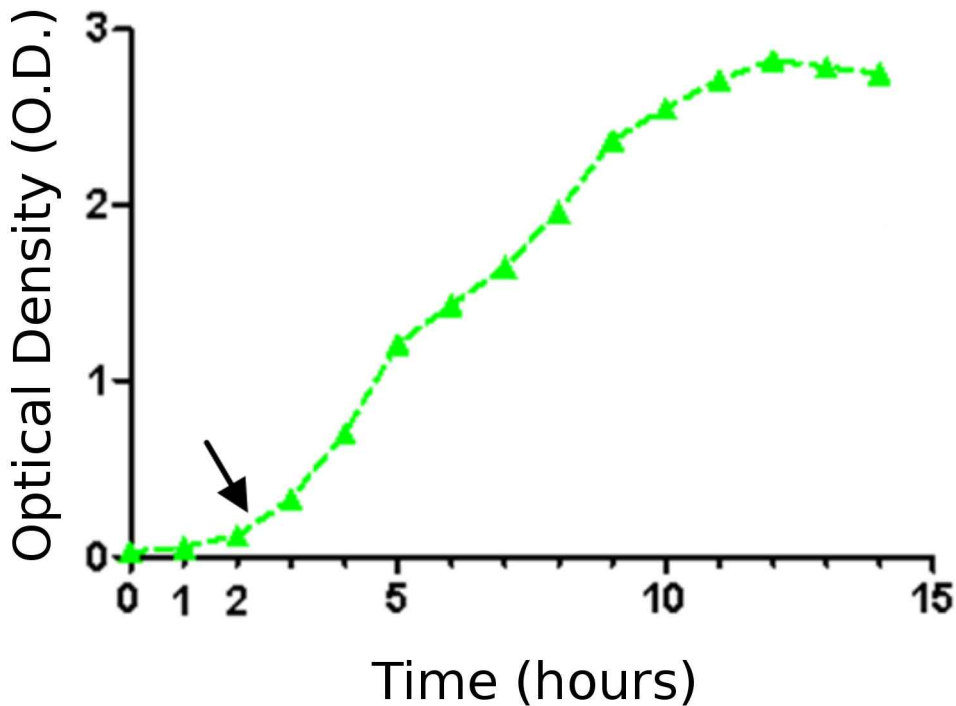

Supplement: Additional file 10: Figure S4 — Growth Curve. Plot showing the growth curve of C. pseudotuberculosis strain 1002 under the control condition and measured at an optical density of 600 nm. Triangles indicate the density values at each hour. The arrow shows the time-point when the stresses were induced. (OD600nm = 0.2) – indicates the beginning of the exponential phase. [file 1471-2164-15-14-S10.pdf]
